# MXene‐Integrated Contact Lens: A Breakthrough in Wearable Eye Protection and Healthcare

**DOI:** 10.1002/smsc.202400628

**Published:** 2025-06-03

**Authors:** Lunjie Hu, Saman Azhari, Hanzhe Zhang, Yuki Matsunaga, Jun Hirotani, Atsushige Ashimori, Kazuhiro Kimura, Takeo Miyake

**Affiliations:** ^1^ Graduate School of Information, Production and Systems Waseda University 2‐7 Hibikino Wakamatsu Kitakyushu Fukuoka 808‐0135 Japan; ^2^ Department of Chemistry Graduate School of Science Nagoya University Furo‐cho Chikusa‐ku Nagoya 464‐8602 Japan; ^3^ Department of Micro Engineering Graduate School of Engineering Kyoto University Kyotodaigaku‐Katsura C3 Nishikyo‐ku Kyoto 615‐8540 Japan; ^4^ Department of Ophthalmology Yamaguchi University 1‐1 Minami‐Kogushi 1‐chome Ube‐shi Yamaguchi 755‐8505 Japan

**Keywords:** 2D conductive films, biocompatibilities, contact lenses, electromagnetic interference shieldings, MXenes

## Abstract

Smart contact lenses with electronic circuits are rapidly advancing for health monitoring and sensing applications, but concerns over electromagnetic (EM) radiation exposure remain. As these devices are near commercialization, protecting the eyes from such radiation is crucial. MXenes (M_*n*+1_X_*n*_T_*x*_, where M is a transition metal, X is carbon and/or nitrogen, and T_*x*_ denotes the functional groups [e.g., —OH, —F, =O, etc.]), a class of 2D transition metal carbides/nitrides, offer exceptional properties such as high conductivity, biocompatibility, and strong EM shielding, making them ideal for preventing radiation‐induced eye diseases like cataracts. Herein, an MXene‐coated contact lens platform that effectively reduces EM radiation exposure while maintaining over 80% visible light transmission, 90% cell viability, and robust shielding capabilities is presented. This approach achieves stable integration of MXene nanosheets on soft contact lenses and mitigates their oxidation degradation. The lens also enhances dehydration protection and demonstrates safety by showing no signs of inflammation or adverse effects in rabbit eyes. These findings highlight MXene‐coated contact lenses as a promising solution for next‐generation wearable technologies and healthcare applications.

## Graphical abstract

This study presents an MXene‐coated smart contact lens that effectively shields the eyes from electromagnetic (EM) radiation while preventing dehydration. The lens maintains high transparency, conductivity, and biocompatibility, ensuring safe and comfortable wear. Experimental results confirm its potential for next‐generation wearable healthcare applications, offering protection against EM interference and improving eye moisture retention.

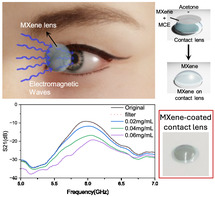

## 1 Introduction

In recent years, advancements in electronics, micro/nanofabrication, information technology, and the Internet of Things (IoT) have spurred significant interest in wearable devices. Among these, contact lenses with electric devices have been developed for various purposes, including vision‐related devices, such as telescope contact lenses,^[^ ^1^ ^]^ alarm systems,^[^ ^2^ ^]^ and augmented reality(AR);^[^ ^3^ ^]^ sensing‐related devices, such as electroretinogram measurement,^[^ ^4^ ^]^ intraocular pressure measurement,^[^ ^5^ ^]^ and glucose measurements;^[^ ^6^ ^]^ therapy‐related devices, such as micropump drug deliver;^[^ ^7^ ^]^ and self‐moisturizing contact lens.^[^ ^8^ ^]^ Many of these systems utilize radio frequency technology for powering or signaling. However, while the electromagnetic interference (EMI) generated by these devices is typically minimal, prolonged exposure to electromagnetic (EM) radiation around the eyes can lead to eye diseases like cataracts.^[^ ^9^, ^10^ ^]^ As IoT technology continues to advance, with an increasing number of wireless devices integrating into our daily lives, the risk of EM radiation exposure to the eyes is also heightened.

MXenes are a class of emerging 2D inorganic compounds composed of atomically thin layers of transition metal carbides, nitrides, or carbonitrides. The general formula of MXene can be expressed as M_*n*+1_X_*n*_T_*x*_, where M is a transition metal, X is carbon and/or nitrogen, and T_*x*_ denotes the functional groups (e.g., —OH, —F, =O, etc.) attached to its surface as a result of the etching MAX (M_*n*+1_AX_*n*_, where n = 1 to 4, and M is an early transition metal, A is an A‐group (mostly groups 13 and 14) element and X is either carbon and/or nitrogen) phase.^[^ ^11^ ^]^ With high electrical conductivity^[^ ^12^, ^13^ ^]^ and solution processability,^[^ ^14^ ^]^ MXenes show tremendous potential in various applications, including Joule heating,^[^ ^15^, ^16^ ^]^ energy conversion/storage,^[^ ^17^, ^18^ ^]^ and EMI shielding.^[^ ^19^ ^]^ For example, unlike traditional dense metal films or hydrophobic graphene, MXene films can provide higher reflective shielding in the microwave frequency range while offering absorption shielding.^[^ ^20^ ^]^ Consequently, MXene‐coated contact lenses have been reported for ocular photothermal therapy^[^ ^21^ ^]^ and for resisting bacterial infection and inflammation,^[^ ^22^ ^]^ but the reported approach lacks stable adhesion of MXene to the fabricated contact lens, not to mention their shortcoming in the prevention of MXene oxidation.

**Figure 1:**
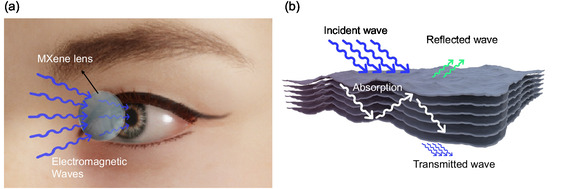
a) Schematic illustration of MXene‐coated contact lens for electromagnetic interference shielding. b) Schematic diagram of MXene electromagnetic interference shielding.

Here, we develop a high‐conductive MXene‐based contact lens platform designed to reduce EM radiation exposure by coating the surface of commercially available contact lenses using Ti_3_C_2_T_*x*_ MXene (**Figure** **1**). Without MXene, EM radiation passes through the contact lens and is absorbed directly by the eyes, potentially causing thermal damage that may lead to cataracts. When MXene is incorporated, some EM radiation is absorbed, while some is reflected by the MXene film due to its multilayered structure on the lens, thereby reducing the EM energy reaching the inner eyes (Figure 1b).^[^ ^23^ ^]^ MXene‐coated contact lenses not only offer high transparency and conductivity but also effectively minimize EM radiation exposure and reduce water evaporation to maintain eye moisture. We believe these MXene‐coated contact lenses present significant potential as a healthcare and bionic platform for future wearable technologies.

## 2 Result and Discussion

### 2.1 Preparation of the MXene‐Coated Contact Lens

**Figure 2:**
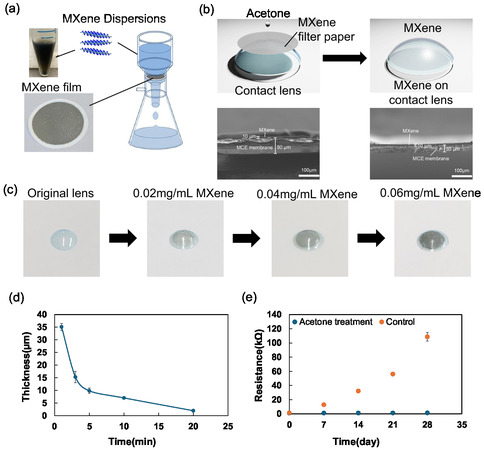
a) Vacuum filtration process used to fabricate MXene thin film on MCE. b) Transfer of the MXene to soft contact lens and cross‐sectional SEM figure of MXene film on MCE membrane and MXene film on acetone‐treated MCE membrane. c) Figures of the original lens, 0.02 mg mL^−1^ MXene‐coated lens, 0.04 mg mL^−1^ MXene‐coated lens, and 0.06 mg mL^−1^ MXene‐coated lens. d) Changes in the thickness of MXene‐covered MCE membrane with the increase in acetone treatment time. e) Resistance of acetone‐treated (MXene film on PEN) and untreated (MXene film on MCE membrane) films under regular environmental conditions within one month. Data are presented as the mean ± SD (*n* = 3).

To coat MXene film on a soft contact lens, we prepared the 0.04 mg mL^−1^ MXene dispersion and filtered 10 mL of the dispersion on mixed cellulose ester (MCE) membranes via vacuum filtration. Once the water was removed, the pump was stopped, and the MXene‐covered membranes were dried in a vacuum oven at 65 °C for at least 24 h before further characterization (**Figure** **2**a). After obtaining the MXene membrane, the membrane was cut into a circle with a diameter of 12 mm and then placed on the commercial contact lens. For the wet transfer process, a small amount of acetone solution was dropped on the MCE‐covered MXene membrane to quickly dissolve the membrane so that the MXene could physically attach to the surface of the contact lens (Figure 2b). Contact lens figures for the original lens, 0.02 mg mL^−1^ MXene‐coated lens, 0.04 mg mL^−1^ MXene‐coated lens, and 0.06 mg mL^−1^ MXene‐coated lens are shown in Figure 2c. This attachment is strong due to the adhesive properties of the dissolved MCE membrane and is less likely to peel off compared to the spray‐coating method. To confirm this, the contact lens fabricated via the wet transfer process and spray coating are placed in water for a month. The MXene film fabricated through the spray coating completely peeled off, but the MXene film fabricated via the wet transfer process stayed intact (Figure S1, Supporting Information). This tendency is due to a thin layer of MCE membrane that cannot completely dissolve after acetone treatment. This can be further observed from Figure 2b, where it is evident that before and after acetone treatment, the film thickness is 80 and 35 μm, respectively. The remaining transparent substance will act like an adhesive layer, holding the MXene membrane on the contact lens and preventing it from peeling off. At the same time, the film thickness gradually decreased with the increase in acetone treatment time, as shown in Figure 2d. To keep the commercial contact lens and film structure intact, the duration of acetone treatment cannot exceed 20 min, so the acetone treatment time was set to 10 min. Since pure acetone is an irritant to the eye,^[^ ^24^ ^]^ we diluted acetone using H_2_O or EtOH (C_2_H_5_OH) to find a minimum acetone concentration that would dissolve the MCE membrane while keeping the treatment time constant (10 min). As shown in Figure S2, Supporting Information, the final membrane thickness is strongly affected by the concentration of acetone in the solution. In addition, EtOH in the solution resulted in nonuniform MXene film formation. The solution with H_2_O did not affect the MXene film uniformity, but the dissolution rate of the MCE membrane was slow. So, there is a potential tradeoff between the dissolution rate and safety. Nonetheless, pure acetone was chosen for the following experiments as in either treatment, post‐washing is required to remove any trace of acetone contamination, while by using pure acetone, we could drastically reduce the dissolution time.

During our experiments, we observed that, in addition to acting as an adhesive layer, the remaining transparent material after the wet transfer process serves as a protective layer to prevent MXene oxidation. To verify its anti‐oxidation ability, we prepared acetone‐treated and untreated films. The acetone‐treated film was the MXene film transferred onto a polyethylene naphthalate (PEN) substrate modified with gold wires via the wet transfer process. In contrast, the untreated film was the MXene membrane obtained after the vacuum filtration step. Both films were exposed to ambient conditions for 1 month, during which the resistance was measured at 7‐day intervals. The gold wires were placed between the MXene layer and substrate to facilitate the measurement after the film adhesion. As shown in Figure 2e, after 1 month, the resistance of the acetone‐treated film increased slightly from 1.22 to 1.46 kΩ, while the resistance of the untreated film increased dramatically from 1.4 to 109 kΩ. The significant rise in resistance of the untreated film is attributed to environmental oxidation. In contrast, the acetone‐treated film showed minimal change in resistance, indicating that the protective layer effectively isolates the MXene from atmospheric oxygen.

### 2.2 Optical Transparency and Conductivity of the MXene Film

**Figure 3:**
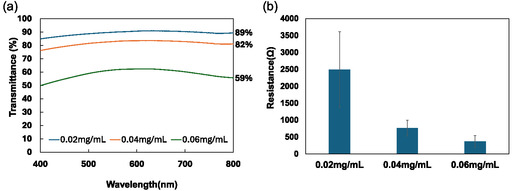
a) Transmittance of 0.02, 0.04, and 0.06 mg mL^−1^ MXene film transferred onto PEN in the visible light region. b) Electrical resistance of 0.02, 0.0 4, and 0.0 6 mg mL^−1^ MXene film on the MCE membrane. Data are presented as the mean ± SD (*n* = 3).

To ensure that the MXene‐coated lens provides clear vision during use, it is crucial that the MXene thin film exhibits high transmittance. We fabricated various MXene films using the vacuum filtration method with different concentrations (0.02, 0.04, and 0.06 mg mL^−1^ of aqueous MXene solution). MXene films were transferred onto a PEN substrate by wet transfer method, and their visible light transmittance was measured, as shown in **Figure** **3**a. The transmittance values for 0.02, 0.04, and 0.06 mg mL^−1^ MXene film on the PEN substrate were 89, 82, and 59%, respectively. The thickness of MXene films was evaluated from cross‐sectional images taken by scanning electron microscope (SEM, S‐3400 N [Hitachi High‐Technologies Corporation, Japan]) (Figure S3, Supporting Information). The film thicknesses for 0.02, 0.04, and 0.06 mg mL^−1^ MXene solution were 1.3, 2.1, and 2.9 μm, respectively. Another important property is the conductivity of the MXene film, as it plays a vital role in effective EM shielding. Therefore, the conductivity of the various MXene films was measured, and as shown in Figure 3b, the resistance values for the films with 0.02, 0.04, and 0.06 mg mL^−1^ MXene concentrations were 2493, 761, and 369 Ω, respectively. As the MXene concentration increases, the film thickness and conductivity also increase. Since the primary goal of the MXene‐coated lens is to maintain clear vision, we aimed for a transmittance level above 80%. Balancing both transmittance and conductivity, we ultimately selected the 0.04 mg mL^−1^ MXene concentration.

### 2.3 Dehydration Protection

**Figure 4:**
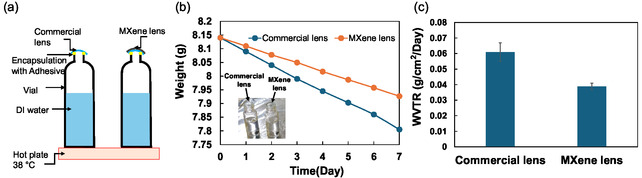
Enhanced dehydration protection by the MXene‐coated contact lens. a) Schematic diagram of the experimental setup for measuring water evaporation rate through the contact lens. b) Weight loss measured on a hot plate at 38 °C for a week. c) WVTR values of the commercial and MXene‐coated contact lens. Data are presented as the mean ± SD (*n* = 3).

By measuring the evaporation rate of water from bottles with a contact lens covering the opening, the dehydration protection effect of the MXene‐coated contact lens was investigated. Small bottle samples were placed on a 38 °C hotplate, and mass loss was measured daily using an electronic balance (**Figure** **4**a). After 7 days, the weights of the commercial lens and MXene‐coated lens decreased by 0.43 and 0.27 g, respectively (Figure 4b). The water vapor transmission rate (WVTR) was calculated by considering the lens size (Figure 4c). The WVTR of the commercial contact lens was 0.061 g cm^−2^ day^−1^. In comparison, the WVTR of the MXene‐coated contact lens was 0.039 g cm^−2^ day^−1^, indicating the potential application of the MXene‐coated contact lens for dehydration protection. Compared to commercial contact lenses, the WVTR of MXene‐coated contact lenses decreased by 36%, possibly due to the multilayer structure of the MXene, demonstrating the positive role of MXene in improving dehydration protection in commercial contact lenses. Also, we evaluated the weight loss of water‐filled vials covered with the MXene‐coated lens of different concentrations (Figure S4, Supporting Information). The WVTRs of the commercial lens were 0.0609 ± 0.006 g cm^−2^ day^−1^. By increasing the concentration of MXene on the lens, the WVTRs decreased to 0.0468 ± 0.0032, 0.0388 ± 0.0021, and 0.0282 ± 0.0021 g cm^−2^ day^−1^ for the 0.02, 0.04, and 0.06 mg mL^−1^ MXene‐coated lens, respectively.

### 2.4 EMI Shielding

**Figure 5:**
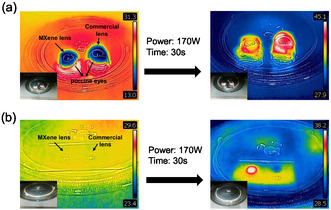
a) Infrared (IR) camera images of the MXene‐coated and commercial contact lens on the porcine eye placed in a microwave oven to test the electromagnetic shielding ability. b) IR camera images showing the elevated temperature of the MXene‐coated lens inside a microwave oven while the temperature of the commercial contact lens is nearly unchanged.

To confirm the EMI shielding effect and durability of MXene‐coated contact lenses, we placed MXene‐coated and commercial contact lenses that were kept in water for a month on freshly thawed porcine eyes. The initial temperature of the porcine eyes was around 13 °C, and then they were placed in a 170 W microwave oven for 30 s. After microwave heating, the temperature of the porcine eyes under the MXene‐coated contact lens rose to around 36 °C, while the temperature of the porcine eyes under the commercial contact lens exceeded 45 °C (**Figure** **5**a). To further investigate the EMI shielding ability of the MXene film, we exposed both contact lenses to strong EM radiation (170 W, for 30 s) inside a microwave oven. Subsequently, thermal imaging was performed using an infrared camera (Figure 5b). The results revealed a rapid increase in temperature for the MXene‐coated contact lens to 38  C, while the temperature of the commercial lens remained relatively stable. This temperature increase in the MXene‐coated lens is attributed to the absorption of EM energy when MXene is exposed to EM radiation, dissipating some of the energy in the form of heat, demonstrating the superior EMI shielding effect of MXene‐coated contact lens and the durability of the fabricated device through the wet transfer process.

**Figure 6:**
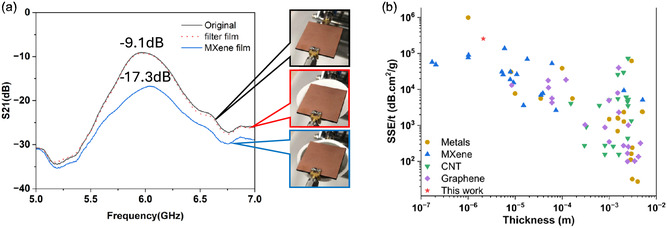
a) S21 data of wireless system without film, with pristine MCE membrane, and with MXene film on MCE membrane. b) Comparison of SSE/t as a function of thickness with previous reports.

To conclude the EMI shielding abilities of MXene‐coated contact lenses and determine the proportion of EM shielding achieved, we employed two microstrip patch antennas with identical resonance frequencies of 5.8 GHz, commonly used in current Wi‐Fi applications. We utilized a vector network analyzer (VNA) to measure S21 (insertion loss), representing energy transmission efficiency. We obtained the EMI shielding ability by comparing the energy transmission efficiency with and without the MXene film between the two antennas. As illustrated in **Figure** **6**a, we measured the S21 values for three states: first, the initial state; second, with a plain MCE membrane in between the two antennas; and third, an MXene‐covered MCE membrane in between the two antennas. It can be observed that the S21 values for the plain MCE membrane and the initial state were both −9.1 dB, indicating that the plain MCE membrane had no EMI shielding effect. However, the S21 value decreased to −17.3 when the MXene‐covered MCE membrane was placed in the middle, representing a decrease of 8.2 dB, indicating that the MXene film can shield ≈85% of the EM radiation. It can be concluded that the EMI shielding effect of the MXene film is significant. Additionally, we compared the EMI shielding effects of 0.02, 0.04, and 0.06 mg mL^−1^ MXene films (Figure S5, Supporting Information), with the S21 values decreasing by 2.8, 8.2, and 12.1 dB, shielding ≈47.5, 85, and 93.8% of the EM radiation, respectively. It can be observed that the EMI shielding effect of the MXene film, similar to the electrical conductivity of MXene, increases with the concentration and thickness of the MXene film. The specific EMI shielding effectiveness (SSE/t, EMI shielding effectiveness divided by density and thickness) was further investigated and compared with the reported works. As shown in Figure 6b, our MXene film exhibits high SSE/t values. The best SSE/t value reaches 257 600 dB cm^2^ g^−1^, which is the highest compared with the previously reported MXene films SSE/t in the same thickness range.^[^ ^25^ ^]^

### 2.5 Biocompatibility

**Figure 7:**
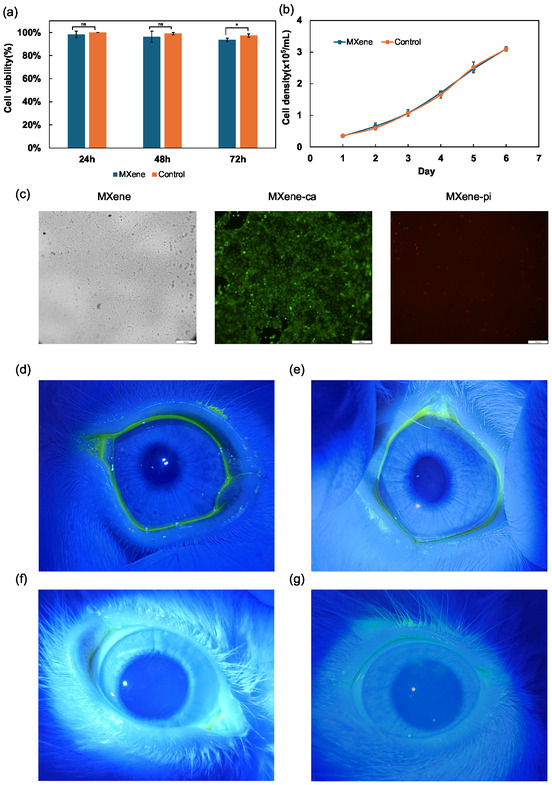
a) Cell viability assay of human corneal cells seeded on the MXene‐coated contact lens and seeded on a dish as control. b) Growth rate for human corneal cells seeded on the MXene film on PEN and seeded on a dish as control. c) MXene film on PEN with unstained cells, cells stained with calcein AM, and propidium iodide. The scale bar is 200 μm. d) Slit lamp micrographs of the corneal fluorescein‐stained left eye of a rabbit. e) Slit lamp micrographs of the corneal fluorescein‐stained right eye of a rabbit. f) Slit lamp micrographs of the corneal fluorescein‐stained left eye of a rabbit after wearing the MXene‐coated contact lens continuously for 10 h. g) Slit lamp micrographs of the corneal fluorescein‐stained right eye of a rabbit after wearing commercial contact lens continuously for 10 h (control experiment). The intensity of fluorescence on the cornea represents the level of corneal damage. Data are presented as the mean ± SD (*n* = 3). Significance level was implied by *, **, ***, and ns for *p* < 0.05, *p* < 0.01, *p* < 0.001, and no significance, respectively.

To ensure the developed contact lens is suitable for daily use, validating its biocompatibility is essential. Assessing the temporal cytotoxicity of the MXene film in human corneal cell (HCE) lines is crucial for detecting potential adverse reactions in vitro. **Figure** **7**a shows the viability assay of HCEs that were seeded on the surface of the MXene film in a culture medium at 37 °C. The viability was calculated by counting the number of live cells stained with calcein AM and dead cells stained with propidium iodide as described in our previous works.^[^ ^26^ ^]^ The cell viability exceeded 94% throughout the entire assay period (72 h) for MXene film. Figure 7b shows nearly identical growth rates for HCEs seeded on the MXene film and seeded on the dish (control), indicating that the MXene membrane does not affect the growth rate of cells. In addition, we confirmed the shape of the cells on the MXene film in Figure 7c. These observations confirm that MXene film is nontoxic, as the majority of the cells remained viable, indicating a negligible risk of corneal inflammation and ensuring the lens is safe for users. This claim was further investigated through in vivo testing using a rabbit model to assess the safety of the lens for daily wear. Corneal fluorescein staining tests were performed, as shown in Figure 7d–g. No signs of corneal abrasions and irritation were observed after wearing the MXene‐coated contact lens for 10 h. We also summarize the changes in the ocular surface overtime after wearing ordinary and MXene contact lenses (Table S1, Supporting Information). Congestion and corneal damage scores inevitably increased overtime in both the first and second tests, but they also increased with the control lens. In contrast, the MXene lens had better overall scores than the controls. Hence, regarding safety, MXene lenses are comparable to normal lenses. These findings demonstrate that our MXene‐based contact lenses do not irritate the cornea and are safe for continuous use.

## 3 Conclusion

In summary, we successfully demonstrated the application of MXene‐coated soft contact lens fabricated using shape‐retaining coatings for EMI shielding and dehydration protection. By transferring MXene films onto a soft contact lens, we achieved high transparency and conductivity. The excellent biocompatibility of MXene materials ensures that these contact lenses can be worn continuously and safely. Furthermore, we confirmed the enhanced dehydration protection provided by the MXene‐coated lens by monitoring the water evaporation rate variation. Testing MXene‐coated contact lenses under strong EM radiation in a microwave oven validated their EM shielding function, showing that MXene can absorb EM energy and release it in the form of thermal radiation, effectively preventing heating of the porcine eyes. In contrast, porcine eyes under commercial contact lenses exhibited significant heating. Furthermore, dual‐antenna S21 measurements confirmed that MXene films can shield ≈85% of EM radiation around 5.8 GHz. These findings suggest that MXene‐coated contact lenses will provide significant potential as a platform for future wearable technologies in healthcare and bionics.

## 4 Experimental Section

4.1

4.1.1

##### Synthesis of Aqueous MXene Dispersion

The aqueous MXene synthesis was based on a previously reported work.^[^ ^27^ ^]^ In short, 40 mg of Ti_3_C_2_T_*x*_ MXene (Japan Material Technologies Corporation), 360 mg of tetramethylammonium reagent, and 22 mL of co‐solvent (tetrahydrofuran/water [H_2_O] [10/1]) were added to a vial and stirred. After stirring at room temperature for 120 h, the mixture was transferred to a 50 mL conical tube. After adding 2‐propanol (15 mL), the mixture was centrifuged at 4,500 rpm for 10 min (Beckman Coulter Allegra X‐30R), and the supernatant was discarded. After adding 20 mL of deionized (DI) water to the sediment, the mixture was shaken by hand for 5 min. To remove non‐delaminated MXene, the mixture was centrifuged at 1,500 rpm for 30 min. The resulting supernatant was centrifuged again at 3,500 rpm for 30 min to remove smaller MXene flakes. Finally, 4 mL of DI water was added to the collected sediment as the MXene dispersion.

##### Fabrication of MXene Films

Three samples, including 0.02, 0.04, and 0.06 mg mL^−1^ of aqueous MXene nanosheet, were prepared. The MXene solutions were filtered through MCE membranes (MF‐Millipore Membrane Filter, 0.05 μm pore size) for 3 min using a vacuum filtration setup. Once the water was removed, the pump was turned off, and the MXene‐covered membranes were dried in a vacuum oven at 65 °C for a minimum of 24 h before further characterization. Upon obtaining the MXene membrane, it was cut into a 12 mm diameter circle using a biopsy punch (Merck Millipore). Then, the circular MXene membrane was placed onto the commercially available soft contact lens (Wavecontact), and a small amount of acetone solution was applied to dissolve the MCE membrane rapidly, facilitating the adhesion of the MXene to the surface of the contact lens. To remove all traces of acetone, the fabricated contact lens was rinsed with DI water for 3 min and then soaked for 24 h in DI water.

##### Evaluation of Biocompatibility

The device was sterilized using ethanol–DI water mixture (70: 30 v v^−1^) for 30 min, rinsed with phosphate‐buffered saline (PBS), and dehydrated using UV irradiation (GL‐15 Toshiba Corporation., Japan) for 1 h. The sterilized sample was placed in a 24‐well plate (Thermo Fisher Scientific., China). HCEs (1 × 10^5^ cells/well) were seeded in a cell medium and subsequently incubated in a humidified incubator maintained at 37 °C with 5% CO_2_ for 24, 48, and 72 h. Trypsin (Cell Science & Technology Institute, Inc., Japan) was used to wash the cells, followed by washing twice and resuspending in PBS. Cell staining was performed by adding 50 μL trypan blue staining solution (Gibco by Thermo Fisher Scientific., Japan) to equal volume of cells, mixing gently, and staining for 3–5 min. A small number of stained cells were counted using a cell‐counting board (Bio‐Rad Laboratories. Inc., USA) to determine the cell viability. The samples with cells were added with calcein‐AM solution (FUJIFILM Wako Pure Chemical Corporation, Japan) and propidium iodide solution (FUJIFILM Wako Pure Chemical Corporation, Japan) separately and then photographed under the IX83 microscope (Olympus Corporation, Japan). For the daily wear safety test, first rabbit was anesthetized via intramuscular injection of a mixture of 0.15 mg Kg^−1^ medetomidine hydrochloride (Domitor; Nippon Zenyaku Kogyo, Japan), 1 mg Kg^−1^ midazolam (Dormicum, Maruishi Pharmaceutical, Japan), and 1.5 mg Kg^−1^ butorphanol tartrate (Betlfal, Meiji Animal Health, Japan). After 10 min, fluorescein sodium was instilled into the sclera under the rabbit's eye using fluorescein ocular examination test papers (0.7 mg [AYUMI Pharmaceutical Corporation, Japan]). Then, slit‐lamp microscopy (NEITZ, Japan) was used to examine the eyes and capture the figures. During the experiment, saline solutions were dropped in the eye every 15–20 min to prevent drying of the eye, general anesthesia was performed every 2–3 h, and rabbits were kept warm with a carpet underneath and a blanket on top. MXene and commercially available contact lenses were worn for 10 h. Slit‐lamp microscopy was repeated after removing the contact lenses to examine the eyes and capture the figures.

##### Fabrication of Microstrip Patch Antenna

The microstrip patch antenna was fabricated on a double‐sided copper‐clad flame retardant 4 (FR‐4) substrate with a copper layer thickness of 35 μm on each side. Its design follows the simple rectangular antenna, with one side as the ground layer, for a working frequency of 5.8 GHz.^[^ ^28^, ^29^ ^]^ The design was simulated using ANSYS HFSS software and fabricated via photolithography and acid etching techniques. The S11 parameters of antennas were measured using VNA (Anritsu‐MS46122B). The S11 parameters of the simulated and fabricated antennas are shown in Figure S6, Supporting Information, while the parameters used to simulate and fabricate the antennas are shown in Table S2, Supporting Information.

##### Statistical Analysis

The experimental data was organized using Microsoft Excel and analyzed using IBM SPSS Statistics version 29.0.2.0. The data were expressed as mean ± standard deviation (SD) and presented without further preprocessing. The resistance measurement, WVTR, cell viability, and growth rate experiments comprised at least three independent experimental batches performed under identical conditions (*n* ≥ 3). For cell viability, statistical analysis was performed by one‐way analysis of variance. In all cases, significance levels (p values) were indicated with asterisks and specific p values were provided in each figure (*p < 0.05, **p < 0.01, ***p < 0.001, and *p* > 0.05 as *ns*).

## Conflict of Interest

The authors declare no conflict of interest.

## Author Contributions

**Lunjie Hu**: data curation (lead); formal analysis (supporting); investigation (supporting); methodology (supporting); and writing—original draft (lead). **Saman Azhari**: conceptualization (equal); data curation (equal); formal analysis (equal); investigation (equal); methodology (equal); project administration (supporting); and writing—review and editing (equal). **Hanzhe Zhang**: data curation (supporting); formal analysis (supporting); investigation (supporting); methodology (supporting); and writing—original draft (supporting). **Yuki Matsunaga**: investigation (supporting); methodology (supporting); and writing—review and editing (supporting). **Jun Hirotani**: conceptualization (supporting); data curation (supporting); funding acquisition (equal); investigation (supporting); methodology (supporting); and writing—review and editing (supporting). **Atsushige Ashimori**: investigation (supporting); methodology (supporting); and writing—review and editing (supporting). **Kazuhiro Kimura**: data curation (supporting); funding acquisition (supporting); investigation (supporting); methodology (supporting); project administration (supporting); and writing—review and editing (supporting). **Takeo Miyake**: conceptualization (lead); formal analysis (lead); funding acquisition (lead); investigation (lead); methodology (lead); project administration (lead); resources (lead); supervision (lead); validation (lead); and writing—review and editing (lead).

## Ethics Approval Statement

The rabbit experiments were approved by the Animal Ethics Committee of the Yamaguchi University Graduate School of Medicine, approval number 41‐003. They were performed in accordance with the ARRIVE guidelines for animal research and the ARVO Statement for the Use of Animals in Ophthalmic and Vision Research.

## Supporting information

Supplementary Material

## Data Availability

The data that support the findings of this study are available from the corresponding author upon reasonable request.
