# Supplementary material for: MXene‐Integrated Contact Lens: A Breakthrough in Wearable Eye Protection and Healthcare
Source: Small Sci. 2025 Jun 3;5(7):2400628. doi: 10.1002/smsc.202400628 (PMC12257883; doi:10.1002/smsc.202400628)
Supplement: Supplementary file 1 — Supplementary Material [file SMSC-5-2400628-s001.pdf]

MXene-Integrated Contact Lens:
A Breakthrough in Wearable Eye Protection and Healthcare

Lunjie Hu, Saman Azhari, Hanzhe Zhang, Yuki Matsunaga, Jun Hirotani, Atsushige Ashimori, Kazuhiro Kimura, and Takeo Miyake[*]

[*] Corresponding author. Tel./Fax: +81-93-692-5158

E-mail address: miyake@waseda.jp. (T. Miyake)

Supporting information

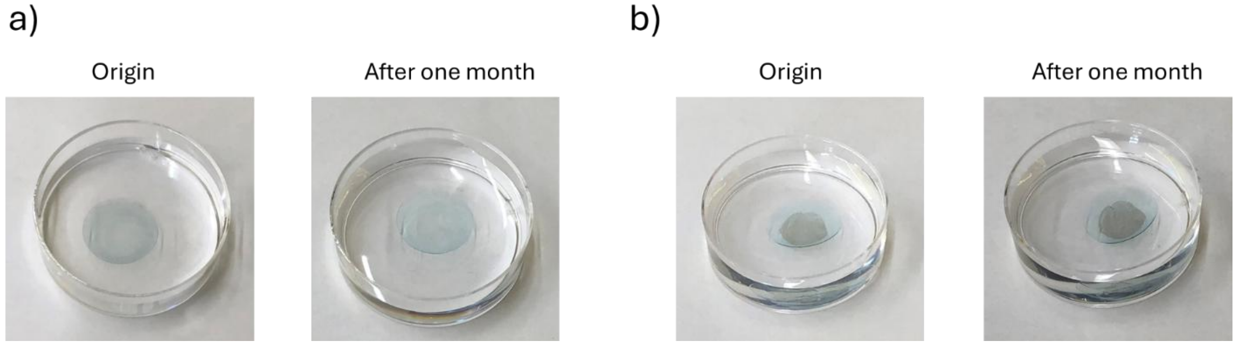

Figure S1. a) MXene spray-coated contact lens in water for 1 month. b) Wet transfer MXene-coated contact lens in water for 1 month.

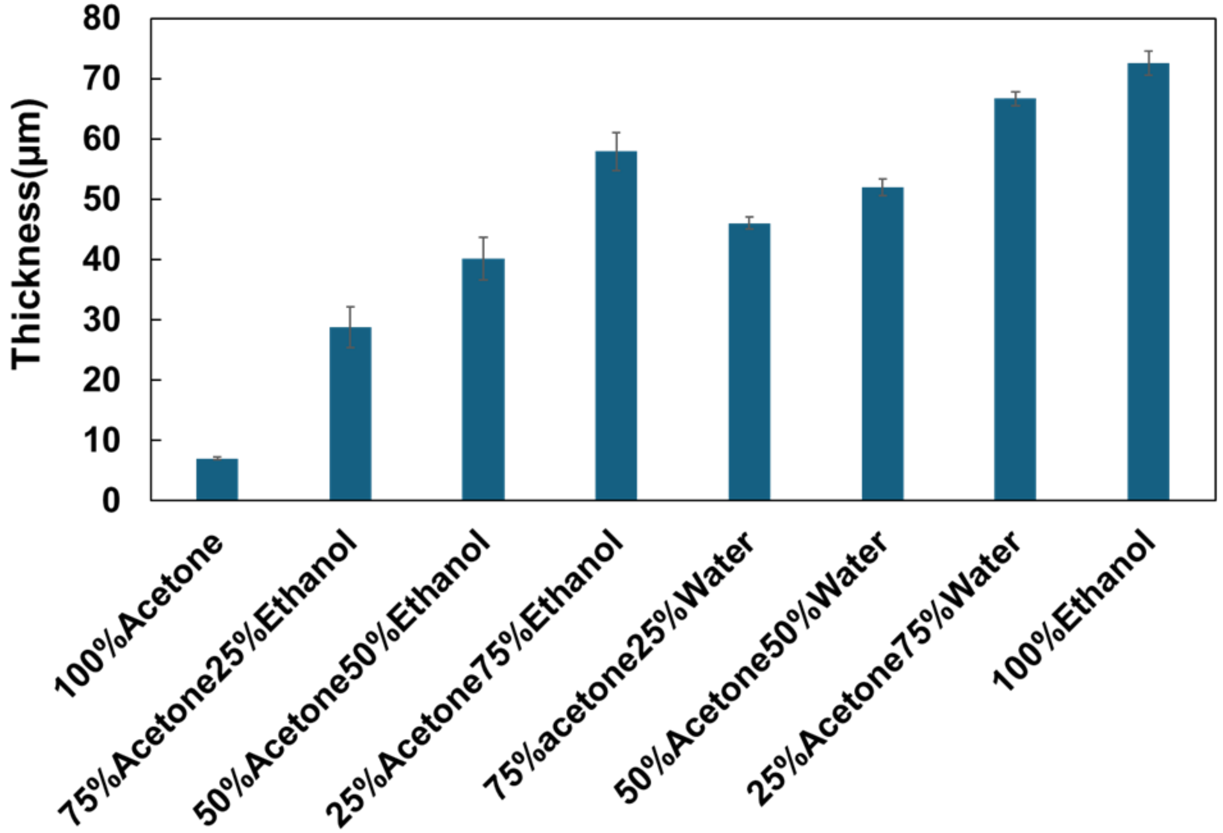

Figure S2. The thickness of the MXene-covered MCE membrane, treated with different solvents after 10 minutes. Data are presented as the mean ± SD (*n*=3).

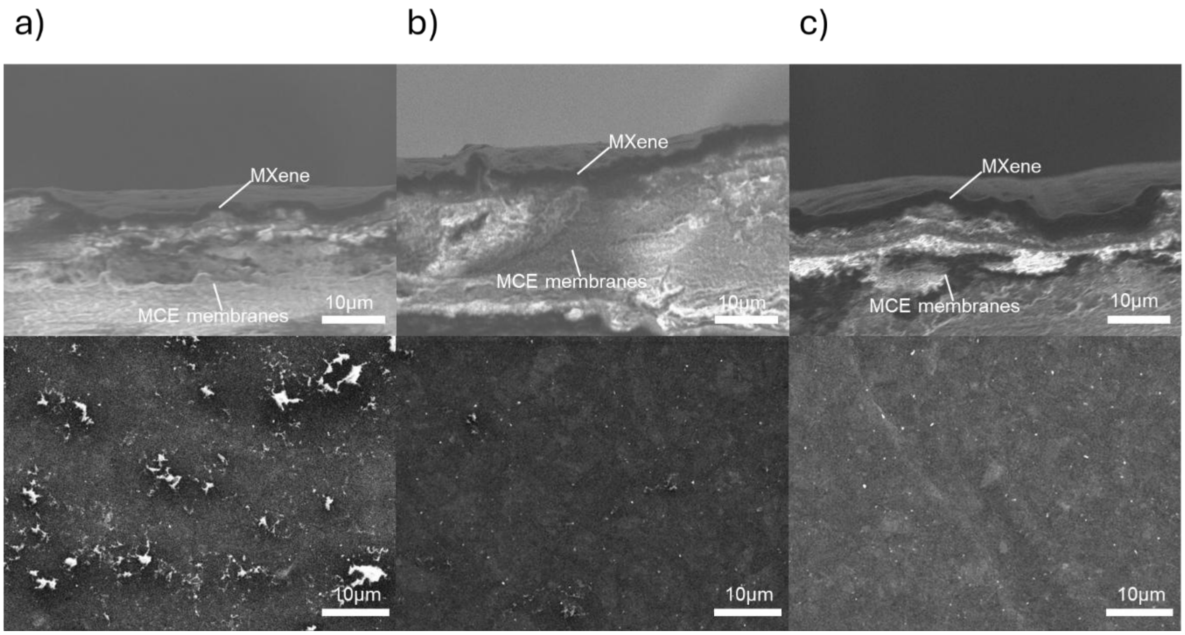

Figure S3. Cross-sectional and top view SEM figure of a) 0.02 mg/mL b) 0.04 mg/mL c) 0.06 mg/mL MXene film on MCE membrane.

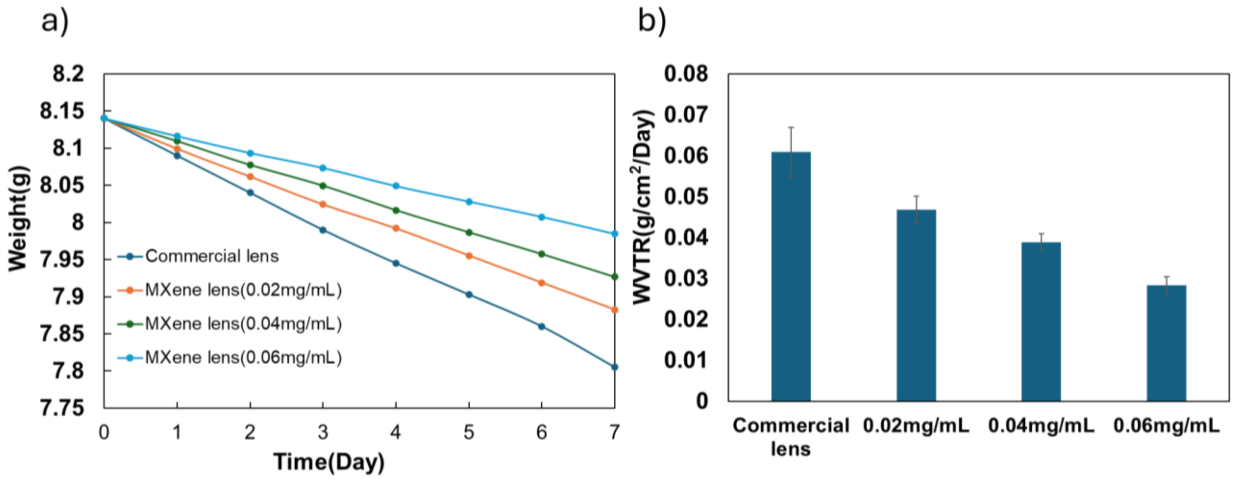

Figure S4. a) Weight loss of water in a container sealed with different MXene-coated contact lens. b) WVTR values calculated from the measured weight loss values. Data are presented as the mean ± SD (*n*=3).

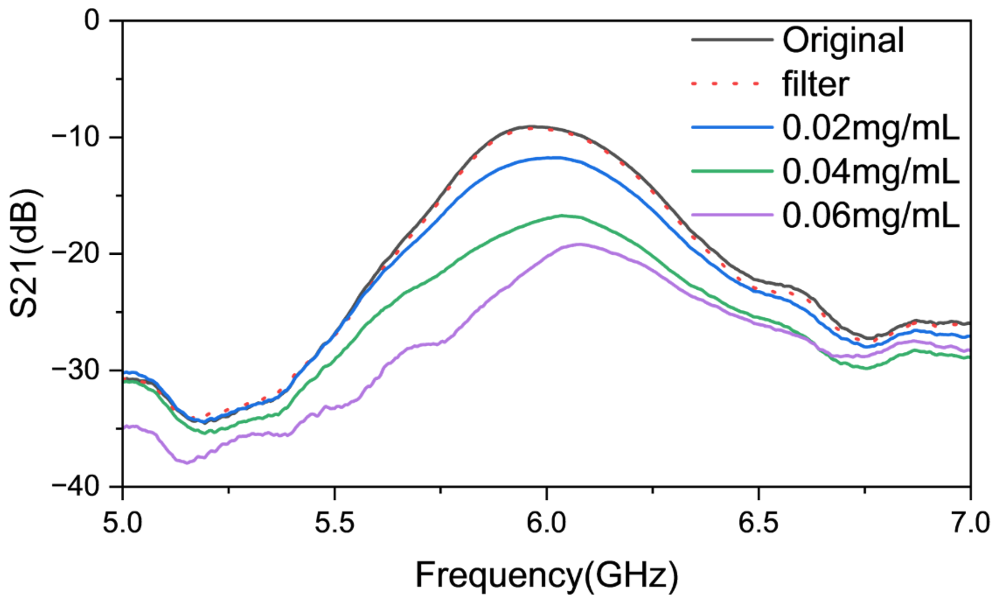

Figure S5. S21 data of wireless system with a different mass of the MXene film on MCE membrane.

		Palpebral conjunctiva				Bulbar conjunctiva		Corneal limbus		Cornea
		Congestion	Swelling	Follicular	Optic disc	Congestion	Edema	Trantas dots	Swelling	Epithelial damage
Normal Lens 1	0h	±	-	-	-	-	-	-	-	±
	10h	++	+	-	-	+++	-	-	-	+
MXene Lens 1	0h	+	-	-	-	+	-	-	-	-
	10h	++	±	-	-	+	-	-	-	±
Normal Lens 2	0h	+	-	-	-	+	-	-	-	-
	10h	+	-	-	-	+	-	-	-	+
MXene Lens 2	0h	+	-	-	-	+	-	-	-	-
	10h	+	-	-	-	+	-	-	-	-

Table S1: Ocular Surface Changes Over Time After Wearing Normal and MXene Lens. This table summarizes ocular surface abnormalities observed at different time points (0h, 10h) after wearing Normal and MXene contact lenses. Evaluated parameters include congestion, swelling, follicular response, and optic disc changes in the palpebral conjunctiva; congestion and edema in the bulbar conjunctiva; Trantas dots at the corneal limbus; and swelling and epithelial damage in the cornea. The severity of each symptom is indicated by symbols: "-" (none), "±" (mild), "+" (moderate), "++" (severe), and "+++" (very severe).

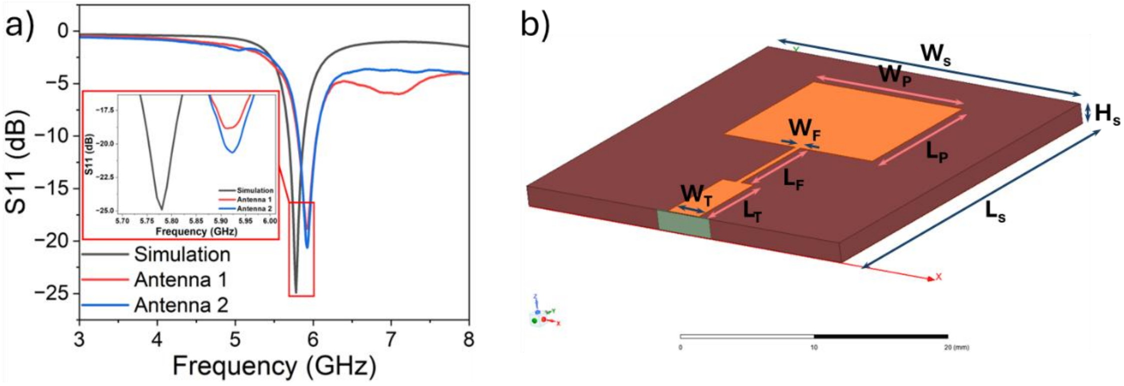

Figure S6. a) The S11 parameter of simulated and fabricated microstrip patch antenna. b) the design and dimensions of the simulated and fabricated microstrip patch antennas.

Dimension	Simulated/Fabricated
Width of transmission line, W_T	3 mm
Length of transmission line, L_T	6.9 mm
Width of feeder, W_F	0.5 mm
Length of feeder, L_F	7.4 mm
Width of patch, W_P	14.3 mm
Length of patch, L_P	11.25 mm
Width of substrate, W_S	30 mm
Length of substrate, L_S	30 mm
Height of substrate, H_S	1.6 mm
Relative permittivity of FR4, ε_r	4.3

Table S2: The simulated parameters of the microstrip patch antenna used for the fabrication.
